# Prevention of sulfide oxidation in waste rock by the addition of lime kiln dust

**DOI:** 10.1007/s11356-019-05846-z

**Published:** 2019-07-04

**Authors:** Elsa Nyström, Hanna Kaasalainen, Lena Alakangas

**Affiliations:** Applied Geochemistry, Luleå University of Technology, 971 87 Luleå, Sweden

**Keywords:** Preventive measures, Acid rock drainage, Sulfidic waste rock, Sulfide oxidation, Waste rock management, Neutralization, By-products, Lime kiln dust

## Abstract

During the operation of a mine, waste rock is often deposited in heaps and usually left under ambient conditions allowing sulfides to oxidize. To focus on waste rock management for preventing acid rock drainage (ARD) formation rather than ARD treatment could avoid its generation and reduce lime consumption, costs, and sludge treatment. Leachates from 10 L laboratory test cells containing sulfide-rich (> 60% pyrite) waste rock with and without the addition of lime kiln dust (LKD) (5 wt.%) were compared to each other to evaluate the LKD’s ability to maintain near neutral pH and reduce the sulfide oxidation. Leaching of solely waste rock generated an acidic leachate (pH < 1.3) with high concentrations of As (21 mg/L), Cu (20 mg/L), Fe (18 g/L), Mn (45 mg/L), Pb (856 μg/L), Sb (967 μg/L), S (17 g/L), and Zn (23 mg/L). Conversely, the addition of 5 wt.% LKD generated and maintained a near neutral pH along with decreasing of metal and metalloid concentrations by more than 99.9%. Decreased concentrations were most pronounced for As, Cu, Pb, and Zn while S was relatively high (100 mg/L) but decreasing throughout the time of leaching. The results from sequential extraction combined with element release, geochemical calculations, and Raman analysis suggest that S concentrations decreased due to decreasing sulfide oxidation rate, which led to gypsum dissolution. The result from this study shows that a limited amount of LKD, corresponding to 4% of the net neutralizing potential of the waste rock, can prevent the acceleration of sulfide oxidation and subsequent release of sulfate, metals, and metalloids but the quantity and long-term stability of secondary minerals formed needs to be evaluated and understood before this method can be applied at a larger scale.

## Introduction

The ability to reduce sulfide oxidation in waste rock after mine closure is a widely researched area, as is, the treatment of acid rock drainage (ARD) formed during operation (Kefeni et al. 2017; INAP 2014; Lottermoser 2010). Less research focuses on the prevention of sulfide oxidation in waste rock during operation. In today’s Sweden, waste rock is stored in heaps close to the pit or underground, thus becoming a part of the hydrological system with water being transported to, through, and from the storage (Amos et al. 2015). The waste rock is thereby left to oxidize under ambient condition until remediation is initiated which is commonly performed during the decommissioning of the mine. During the operation of a mine, formed ARD is conventionally treated actively with alkaline material in an attempt to raise the pH allowing for the precipitation of metals in the formation of sludge (Brown et al. 2002; Younger et al. 2002). Active treatment focuses on treating the symptoms from sulfide oxidation rather than concentrating on preventing the oxidation itself. If measures were put on treating the waste rock rather than the formed ARD, its generation could be mitigated or at least limited, and the consumption of alkaline material could be reduced along with costs for the subsequent sludge treatment.

Sulfide passivation or microencapsulation is an alternative inhibition technique (compare to cover systems, desulfurization, or bacteria inhibition) for controlling ARD formation. Sulfide passivation is described as a chemically inert coating on the sulfidic surface, capable of protecting the sulfidic core from attacks of O_2_ and Fe^3+^ (Zhang and Evangelou 1998). Several additives that could enhance surface coatings have been studied, both organic and inorganic, out of which the most common ones ought to be silica (Fan et al. 2017; Kang et al. 2016; Kollias et al. 2015; Evangelou 1995) or permanganate solutions (Ji et al. 2012; Misra et al. 2006; De Vries 1996). If successful, passivation is considered a low-cost prevention technique, especially compared with traditional mine drainage treatments using alkaline additives (Sahoo et al. 2013a). However, most of the materials studied for passivation are either too expensive or potentially harmful to the environment (Sahoo et al. 2013b) and almost exclusively focus on tailings rather than waste rock. Thus, there is a need to find cost-effective materials able to passivate sulfide surfaces in waste rock in a long-term perspective.

During the last decade, alternative materials such as alkaline secondary raw materials have been studied based on the assumption that passivation can be achieved by maintaining near-neutral pH in the sulfidic mine waste. The theory is related to studies performed by Huminicki and Rimstidt (2009) who found that sulfide oxidation at near-neutral pH, in the presence of sufficient alkalinity, will promote precipitation of secondary minerals such as hydrous ferric oxides (HFO) on the sulfide surface, growing thicker with time, and when thick enough, will prevent the sulfides from further oxidation. Unlike other methods, this creates a self-healing system independent of additives in the long-term. One of the most extensively studied material is fly ash from coal combustion (Sahoo et al. 2013b; Yeheyis et al. 2009; Pérez-López et al. 2007, 2009). However, these studies are based on a relatively high fraction of alkaline industrial residues compared with mine waste and therefore most applicable when the residue originates in the vicinity of the mine. The possibility for this design is unfortunately currently limited in Sweden due to transport costs, which account for the majority of such treatment design. Furthermore, the goal for the mining industry is to limit the total amount of wastes stored at the mine site. There is thus a need for alternative materials, with properties that limit the addition amounts (≤ 5 wt.%) to consider economic and space perspective. Several tests of different secondary raw materials’ suitability for passivation purposes resulted in the selection of lime kiln dust (LKD) (Nyström et al. 2019) as an alternative additive. Results from 52 weeks of leaching of sulfide-rich waste rock covered with a thin layer of LKD (5 wt.%) showed a substantial decrease in acid, metals, and metalloids. However, in this study, the aim was to evaluate the long-term (109 weeks) leaching of waste rock and LKD and the capture of trace elements, in order to inhibit the sulfide surfaces and thereby reduce the sulfide oxidation.

## Materials and methods

### Waste rock

The waste rock originated from one of Boliden Mineral AB’s Zn-Cu-Au-Ag open pit mines in northern Sweden. The mine is a volcanic-associated massive sulfide ore deposited at the bottom of the sea approximately 1.89 billion years ago and is part of the so-called Skellefte group (Montelius 2005).

As previously described, the success of passivation of sulfide surfaces by the formation of secondary minerals highly relies on the ability for sulfides to oxidize under near-neutral pH. To meet one of these requirements, waste rock was selectively chosen based on sulfur content, previously described by Alakangas et al. (2013) and Nyström et al. (2019) and included screening of waste rock piles with a handheld X-ray fluorescence (XRF) of the brand Olympus Innov-x systems, USA, for selectively choosing waste rock with high sulfur content. Alakangas et al. (2013) characterized the waste rock for its major and minor elements showing that it had an average sulfur content of 30%. Nyström et al. (2019) characterized the waste rock for its major mineral content showing that it was dominated by pyrite and quartz (17%) with smaller amounts of sericite (6%), chlorite (4%), and calcite (1%). Approximately 99% of the sulfide content in the waste rock consisted of pyrite with the remaining 1% comprising of, e.g., chalcopyrite, bournonite, sphalerite, arsenopyrite, and pyrrhotite (in descending order of abundance). Moreover, the waste rock showed limited neutralizing ability correlated to the abundance of pyrite and scarcity of buffering minerals.

### Lime kiln dust

Lime kiln dust (LKD) was selected as the alkaline source and is a secondarily formed raw material generated from the manufacturing of quicklime where limestone is added to a rotary kiln and heated to a temperature up to 1200–1300 °C. During the combustion of the limestone, flue gasses are collected and stripped of dust in an electrostatic precipitator. LKD is essentially dust but can be mixed with limestone of varying quality and quantity to make niche products. Nordkalk supplied the LKD, which was mixed with crushed limestone too fine for the kiln.

A previous study by Nyström et al. (2019) showed that the LKD consisted of anhydrite, calcite, gypsum, Mg-rich calcite, Na-rich sylvine, portlandite, and quicklime. Moreover, the study showed that the LKD, compared with many other secondary raw materials, was a rather pure material with low content of easily water-soluble minerals and generally low leachability of trace metals and metalloids such as As (0.08 μg/L), Cu (< 0.5 μg/L), Mn (0.5 μg/L), Pb (0.1 μg/L), Sb (0.08 μg/L), and Zn (< 2 μg/L). The LKD had, before sampling, been stored in piles outdoors, which likely hydrated and re-carbonated the quicklime in the LKD (Nyström et al. 2019).

#### Chemical content

Two samples of LKD were screened of more than 70 elements performed by using inductively coupled plasma mass spectroscopy (ICP-MS) by the SVEDAC-accredited ALS Scandinavia laboratory in Luleå, Sweden. Total element concentrations were analyzed after lithium borate fusion and digestion by nitric, hydrochloric, and hydrofluoric acid.

#### Mineralogical content

The mineralogical study performed by using X-ray powder diffraction (XRD) (Nyström et al. 2019) was complemented by quantitative mineralogical characterization with thermogravimetric measurements using a NETZSCH STA 409 C/CD. Heating was performed up to 1000 °C in inert Ar gas. The quicklime content was estimated using the “sugar rapid method” (ASTM 2011).

### Experimental setup

**Fig. 1:**
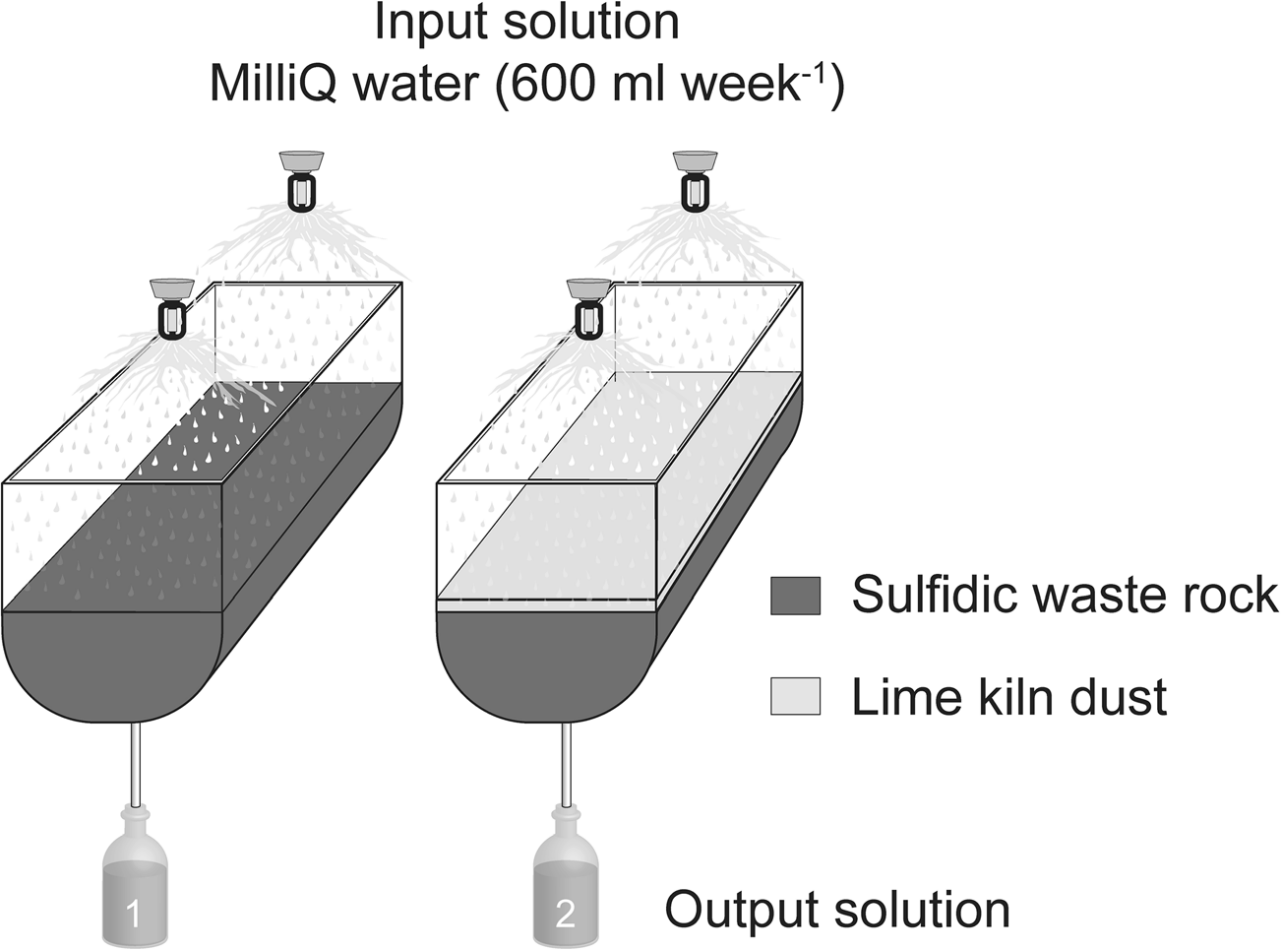
Experimental design of small-scale test cells filled with (1) sulfidic waste rock, (2) sulfidic waste rock, and 5 wt.% aged lime kiln dust

**Fig. 2:**
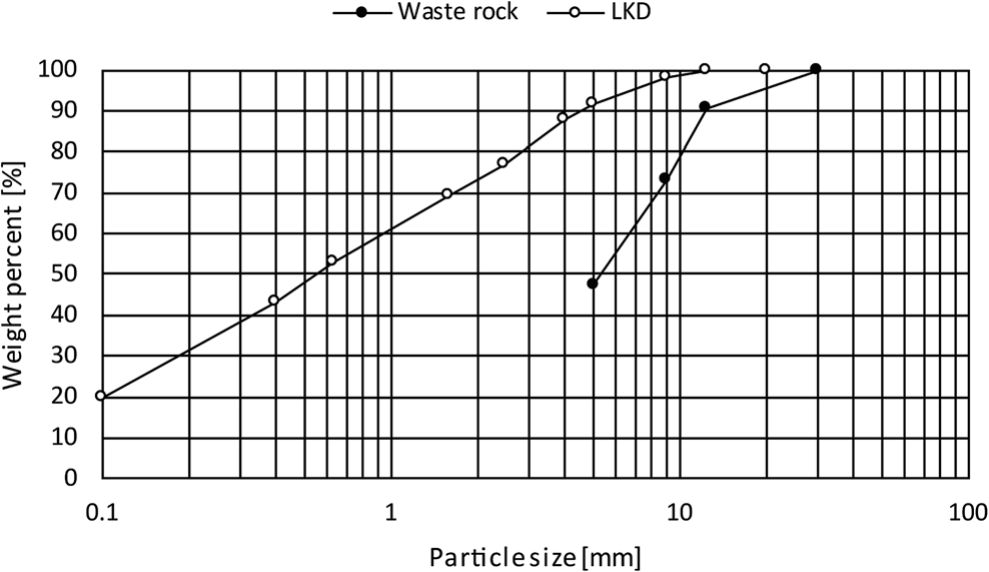
Particle size distribution of the waste rock and the aged lime kiln dust (LKD)

Kinetic testing was conducted in small-scale test cells with the aim to reproduce the oxidation conditions of waste rock and promote secondary mineral formation for passivation of the sulfidic surfaces. The experimental design deviated from column testing often used, such as the AMIRA P387 ARD Handbook (Smart et al. 2002), due to previously mentioned desire to study secondary mineral formation. It has been shown that precipitation of, e.g., HFO is predominant in the interface between the mine waste and alkaline source (Pérez-López et al. 2007; Sahoo et al. 2013b) and for this reason, it was desirable to increase the cells’ surface area. The experimental design consisted of high-density polyethylene cells with surface areas of 513 cm^2^ and a total volume of 10 L (Fig. 1). Four cells were constructed, of which two are presented herein. The cells were irrigated on a weekly basis with 600 mL of MQ water (0.05 μS/cm) corresponding to average annual precipitation in the area and equivalent to a yearly L/S ratio of approximately 4. The bottom of each cell was lined with geotextile to avoid clogging of the tap. Cell 1 was a control cell filled with solely waste rock (7.55 kg) and acted as a reference displaying the evolution of ARD production without any preventive measures for passivation. Cell 2 was filled with waste rock, and after 8 weeks of leaching, a layer of LKD (377 g), corresponding to 5 wt.%, was added on top. A comparison of the leaching behavior of the waste rock during the initial 8 weeks of leaching was performed by Nyström et al. (2019) and revealed important similarities in pH and EC amongst the cells before extra material addition. It was assumed that the two cells presented herein were comparable when the LKD was added on top of the waste rock in cell 2. The waste rock was re-sieved after 3 weeks of leaching due to clogging in the system, which removed most of the fines leaving a 5–30-mm waste rock fraction (Nyström et al. 2019). A more detailed description of the waste rock size variations is seen in Fig. 2.

### Sampling and analysis of leachates

Leachates were collected from the tap located at the bottom front of the cell on a weekly basis the day after irrigation. The collected leachates were measured for pH, electrical conductivity (EC), and temperature in connection with the sampling and in closed containers to avoid exposure to air. The pH and EC were measured using a WTW Multi 3420 multimeter equipped with Sentix® 940 (pH) and TetraCon® 925 (EC) electrodes. Leachate samples were filtrated through a 0.22-μm nitrocellulose membrane into high-density polyethylene bottles using vacuum filtration. The filter equipment was washed with 5% nitric acid, and the filters in 5% acetic acid. Filtrated samples were stored cold (4 °C) and in darkness until analysis. Before analysis, the samples were acidified with 1 mL nitric acid (suprapur) per 100 mL sample. Selected samples were analyzed for major and trace element composition using inductively coupled plasma atomic emission spectroscopy (ICP-AES) and inductively coupled plasma sector field mass spectrometry (ICP-SFMS) at the SVEDAC-accredited laboratory ALS Scandinavia in Luleå. The analysis was either performed according to US EPA Method 200.7 (modified) and 200.8 (modified) or by quantitative screening analysis for over 70 elements.

### Geochemical and mass calculations

Geochemical calculations, including aqueous species distribution, and mineral saturation state, were carried out using PHREEQC version 3.4.0 (Parkhurst and Appelo 1999) using the *wateq4f.dat* thermodynamic database (Ball and Nordstrom 1991). Ion imbalance for considered samples was within ± 14%. The solubility constants of more ordered ferrihydrites, namely 2- and 6-line, were used as reported by Stefánsson (2007), while, for schwertmannite, the solubility constant from Majzlan et al. (2004), originating from Bigham et al. (1996), was considered.

### Solid phases

Grab solid samples were taken before leaching (initially) and from the two cells at two separate occasions year one and two (week 52, 103). Samples were taken approximately 2 cm down into the waste rock profile (8 cm in total) and dried at < 30 °C for a total of 5 h before subjected to small amounts of high-pressured air to remove any excess LKD. Sequential extraction according to Dold (2003) was used to evaluate element distribution in different mineral associated phases as leaching prolonged. Surface analysis was performed using a ZEISS SteREO V.8 stereomicroscope which was used for optical imaging followed by a ZEISS Gemini Merlin scanning electron microscope (SEM) for secondary electron imaging. Mineral identification was performed with Raman analysis (532-nm laser excitation) using a Senterra Raman spectrometer connected to an Olympus BX microscope. For primary minerals, the laser power was set to 2 mW with an exposure time of 1 s with 5 scans and a pixel size of 1 μm. For secondary minerals, the laser power was set to 0.2 mW with an exposure time of 60 s with 3 scans and a pixel size of 1 μm.

## Results and discussion

During the operation of a mine, waste rock is commonly left under ambient conditions for tens of years before preventive measures, such as dry or wet cover, are applied. During the period from excavated to remediated, sulfides present in the waste rock are allowed to oxidize which can have long-term negative effects on the environment. Acid mine drainage generally develops in waste rock heaps where the neutralization capacity, mainly in the form of carbonate minerals, is depleted or low due to ongoing oxidation of sulfide minerals. The aim of this study was to investigate whether a limited amount of LKD to partially oxidized waste rock could enable precipitation of secondary minerals on the reactive surfaces and thereby hinder the sulfide oxidation from accelerating.

### Characteristics of lime kiln dust

**Fig. 3:**
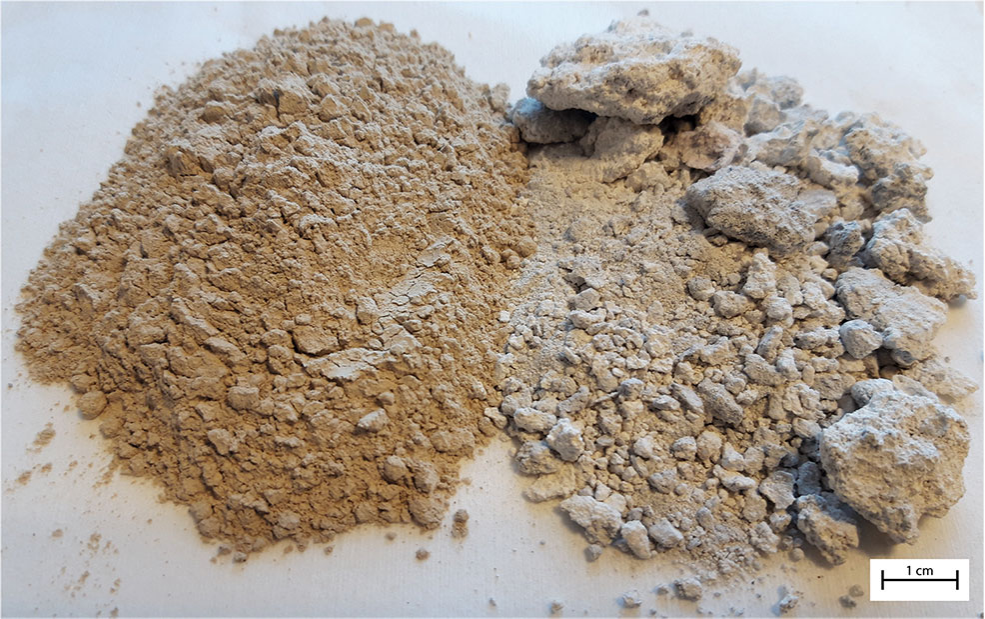
Photo of fresh (left) and aged (right) lime kiln dust

As previously described, LKD has been found suitable to create and maintain a near-neutral leachate pH for 52 weeks (Nyström et al. 2019). Complemented mineralogical studies were performed in an attempt to quantify the mineralogical content of the LKD to assess its suitability to maintain near-neutral pH in the long-term perspective as well as to examine its impact on the leachate quality. Results from mineralogical studies using thermogravimetry can be seen in Table 1 and are estimated based on thermogravimetric curves as described by Földvári (2011). The measurements confirm previous results obtained by Nyström et al. (2019) that the LKD consisted of mainly calcite with smaller amounts of Mg-rich calcite, slaked lime, gypsum, and quicklime. The Ca-rich minerals identified can explain approximately 84% of the Ca content of the LKD (Table 2) with the deviation explained by the heterogeneity of the LKD as previously reported by Nyström et al. (2019). However, it cannot be excluded that the LKD can contain traces of other unidentified minerals. The high calcite content together with the relatively low leachability of trace metals and metalloids is believed to result from storing the material outside. Visual comparison of fresh LKD shows that mixing the LKD with finely crushed limestone followed by storing causes the material to ball up into porous aggregates (Fig. 3) generating a material varying in size from < 0.01–20 mm (Fig. 2) compared with fresh LKD where 100% < 2 mm. Storing the material and thereby letting it leach for some time before adding to waste rock is believed to be positive from several aspects. Several of the easily soluble salts (such as sylvine) present in the LKD have time to dissolve lowering, e.g., the Cl content. Moreover, fresh LKD has been shown to contain up to 40% quicklime whereas the stored LKD herein was found to contain only 1%. As previously described, it has been shown that a near-neutral pH with sufficient alkalinity is needed to generate secondary precipitates capable of passivating sulfide surfaces (Huminicki and Rimstidt 2009). Although calcite dissolution may be sufficient to generate required pH in some wastes, such reactive wastes as the one used herein would require higher alkalinity to increase the pH to necessary levels. Quicklime is easily hydrated, generating high alkalinity leachate in the short-term perspective, which can be a good complement to the more slowly dissolving calcite. However, if the quicklime content is high, as observed in the fresh LKD, the hydration can result in hardening of the material, making it less reactive and not as useful for inhibition purposes (unpubl. data). Therefore, storage of LKD and allowing re-carbonation is believed to improve the material for the type of application described herein. It cannot be determined if storing the LKD would be positive when used in other applications or if other secondary raw materials, such as bark fly ash, would benefit from similar pre-treatment method to rid the material from, e.g., excess salt content.

**Table 1:** Quantitative mineralogical composition of aged lime kiln dust (LKD) determined by thermogravimetry. Quicklime content determined with “sugar rapid” method (ASTM 2011). Anhydrite and sylvine content was estimated based on S and Cl concentrations respectively in the LKD

Mineral	%
Calcite	85
Mg-rich calcite	8
Gypsum	2
Portlandite	2
Quicklime	1
Anhydrite	2
Na-rich sylvine	< 0.2

**Table 2:** Abundance of selected elements in the waste rock, fresh and aged lime kiln dust (LKD). Waste rock is presented as the average of screening and results reported by Alakangas et al. (2013) and Nyström et al. (2019)

	Average	LKD		Average
%	Waste rock	Fresh	Aged	Earth’s crust^1^
Al	2	2	0.3	8
Ca	0.8	52	46	3
Cl	0.009	0.1	< 0.1	0.05
F	0.132	0.06	0.007	0.05
Fe	32	1	0.4	5
K	0.4	0.7	0.02	2.6
Mg	0.5	0.9	0.7	2.1
Na	0.3	0.2	0.02	2.4
P	0.005	0.06	0.003	0.1
S_total_	30	1	0.04	0.05
Si	9	3	0.7	27
Ti	0.03	0.09	0.02	0.5
mg/kg				
As	217	8	0.8	2
Ag	1	2	0.02	0.07
Au	< 0.01	0.009	0.01	0.004
B	5	42	18	10
Ba	65	329	22	430
Be	0.2	2	0.1	3
Bi	0.2	0.8	0.03	0.2
Br	< 5	4	n.a	3
Cd	0.3	4	0.09	0.18
Ce	12	21	7	45
Co	2	7	0.9	25
Cr	16	23	6.9	200
Cs	0.2	5	0.098	2
Cu	19	31	2	60
Dy	1.4	2	1	4.5
Er	0.9	0.9	0.4	
Eu	0.7	0.4	0.2	1.2
Ga	5	5	1.0	17
Gd	1	1	0.8	7
Ge	0.4	1	0.1	15
Hf	2	1	0.2	
Hg	17	0.009	0.05	0.08
Ho	0.3	0.3	0.1	
I	< 0.5	6		0.5
In	< 0.2	n.a		0.1
Ir	< 0.01	0.0006	0.0005	0.001
La	6	10	4	6.5–100
Li	15	25	3	30
Lu	0.1	0.1	0.05	0.9
Mn	189	205	178	900
Mo	3	3	0.2	2
Nb	1	4	1	20
Nd	6	9	4	25
Ni	3	25	3	80
Os	< 0.01	0.0002	0.001	
Pb	22	32	3	16
Pd	< 0.1	0.1	0.1	0.01
Pr	2	2	0.9	
Pt	< 0.01	0.002	0.0026	0.005
Rb	8	35	2	120
Re	< 0.01	0.01	0.0005	0.001
Rh	< 0.01	0.003	0.009	
Ru	< 0.01	0.0001	< 0.00002	
Sb	38	3	0.09	0.15–1
Sc	5	3	0.8	5–22
Se	<1	3	0.5	0.09
Sm	1	2	0.8	7
Sn	0.5	2	0.3	2.5
Sr	29	588	168	350
Ta	< 0.01	0.4	0.05	2
Tb	0.2	0.2	0.1	
Te	0.1	0.2	< 0.00005	0.00036–0.01
Th	1	5	0.8	10
Tl	26	0.7	0.05	1
Tm	0.1	0.1	0.05	
U	1	9	1	3
V	15	3	0.1	150
W	2	39	31	1
Y	8	11	5	30
Yb	0.9	0.8	0.4	3
Zn	68	105	25	70
Zr	50	40	9	160

^1^Krauskopf and Bird 1995

### Metal and metalloid release

The net neutralization potential of the waste rock has previously been measured to − 946 kg CaCO_3_/ton waste rock, displaying a great shortage of carbonate minerals required for neutralization (Nyström et al. 2017). In addition, the amount of mineral exposed for neutralization can be limited.

**Fig. 4:**
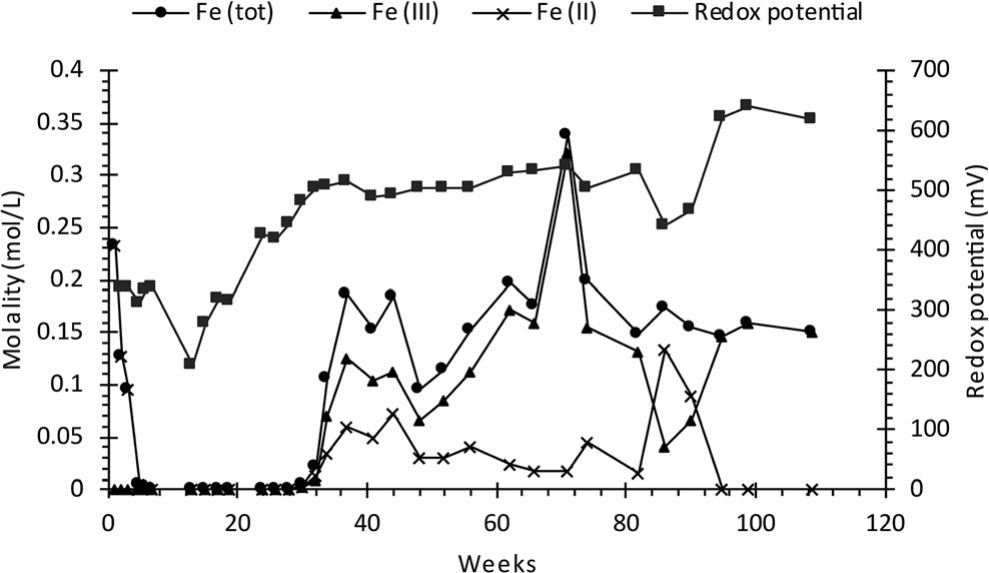
Redox potential and concentration of total Fe, as a function of time in the leaching of waste rock (cell 1). Speciation of Fe was based on geochemical calculations using the *wateq4f.dat* thermodynamic database in the geochemical model PHREEQC

**Fig. 5:**
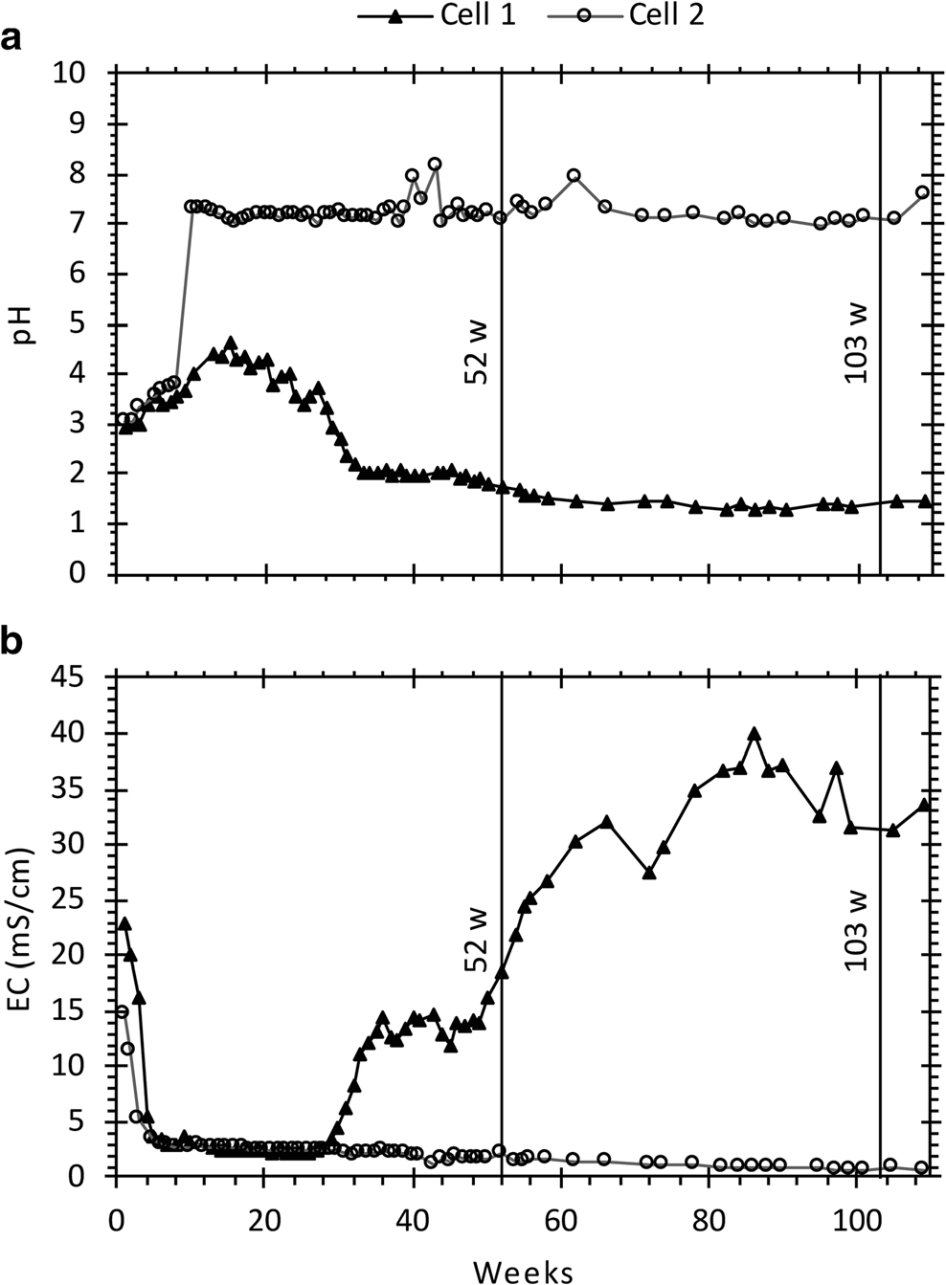
Variations in **a** pH and **b** electrical conductivity, as a function of time in the leaching of waste rock (cell1) and waste rock covered with 5 wt.% aged lime kiln dust (cell2)

Pyrite oxidation is a complex process since, at low pH (< 3.5), ferric iron generally dominates which together with *Acidithiobacillus ferrooxidans* accelerates the oxidation of pyrite and subsequent generation of ARD (Nordstrom 2011; Singer and Stumm 1970). Herein, accelerated sulfide oxidation defined as when Fe^3+^ dominates Fe^2+^ in the leachate (Fig. 4). As seen in Fig. 5, not treating the waste rock in cell 1 resulted in a decreasing pH (< 1.5) and increasing EC (> 30 mS/cm) which, at the end of leaching period, resulted in high concentrations of, e.g., Al (220 mg/L), Fe (8.1 g/L), and S (8.4 g/L). Moreover, trace elements such as As (4.9 mg/L), Cu (101 μg/L), Pb (564 μg/L), Sb (168 μg/L), and Zn (4.5 mg/L) were found at elevated concentrations. Once sulfide oxidation is established and ARD starts to form, it is extremely difficult to control and therefore, preventive measures at an early stage of mining are of utmost importance (Dold 2017).

**Fig. 6:**
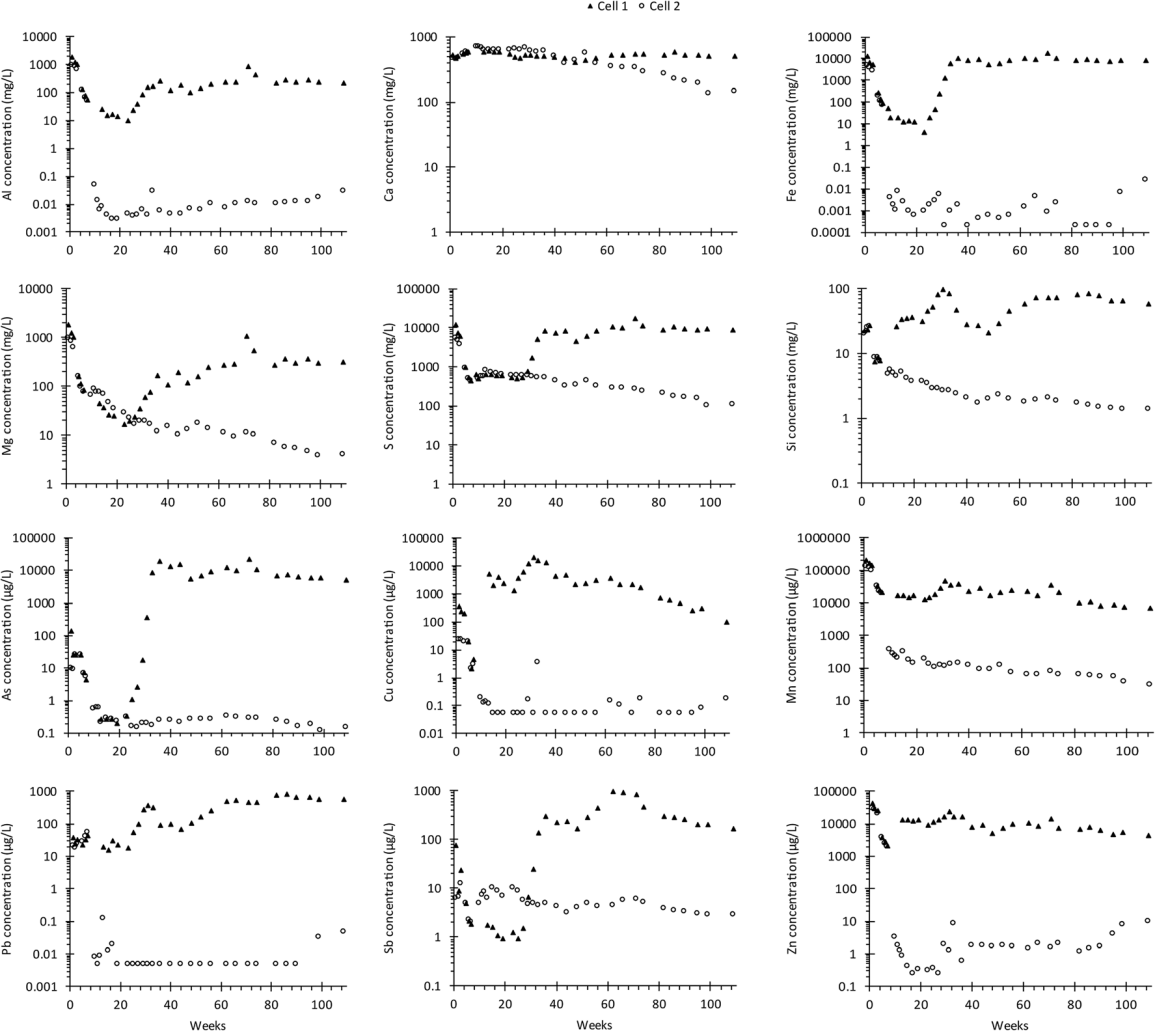
Concentrations of selected main and trace elements in leachates produced from leaching of waste rock (cell 1) and waste rock with 5 wt.% aged lime kiln dust added on top (cell 2)

An addition of 5 wt.% LKD corresponds to less than 4% of the total neutralization potential required to reach a positive net neutralization potential in the waste rock which not only limits the amount of material required to neutralize ARD but also limits the total amount of material stored at the site. As shown in Fig. 5, the addition of LKD to cell 2 successfully managed to create and maintain a near-neutral pH in the leachate for 2 years with no signs of accelerated sulfide oxidation or ARD formation. Instead, concentrations of, e.g., Al (0.03 mg/L) and Fe (0.03 mg/L) along with As (0.15 μg/L), Cu (0.17 μg/L), Pb (0.05 μg/L), Sb (2.8 μg/L), and Zn (9.9 μg/L) were lowered throughout the time of leaching (Fig. 6).

Since sulfide oxidation was not accelerated, lower concentrations of elements were released from cell 2 than from cell 1, and by creating a near-neutral pH environment, metals and metalloids were captured, which largely immobilized their transport. Secondary minerals can precipitate in-between minerals or on mineral surfaces. In order to prevent sulfide oxidation, secondary minerals such as HFO need to grow into a thick rim on the sulfide surface to hinder oxygen intrusion, which takes time (Huminicki and Rimstidt 2009). Therefore, it is not sure that low concentrations of metals and metalloids in the leachate were an effect of decreased sulfide oxidation or decreasing due to their capture in between minerals.

Leachate quality time-series combined with geochemical calculations and sequential extraction was used to describe geochemical reactions occurring in the waste rock covered with 5 wt.% LKD. The method used for sequential extraction (Dold 2003) targets Cu-sulfides and did not generate satisfactory results in the last three steps. Therefore, only the first four steps were considered and compared with the whole rock composition from NaOH-fusion. The remaining steps target both sulfide and silicate minerals. The aim of sequential extraction in this article was to determine the capture of trace elements in steps I–IV.

**Fig. 7:**
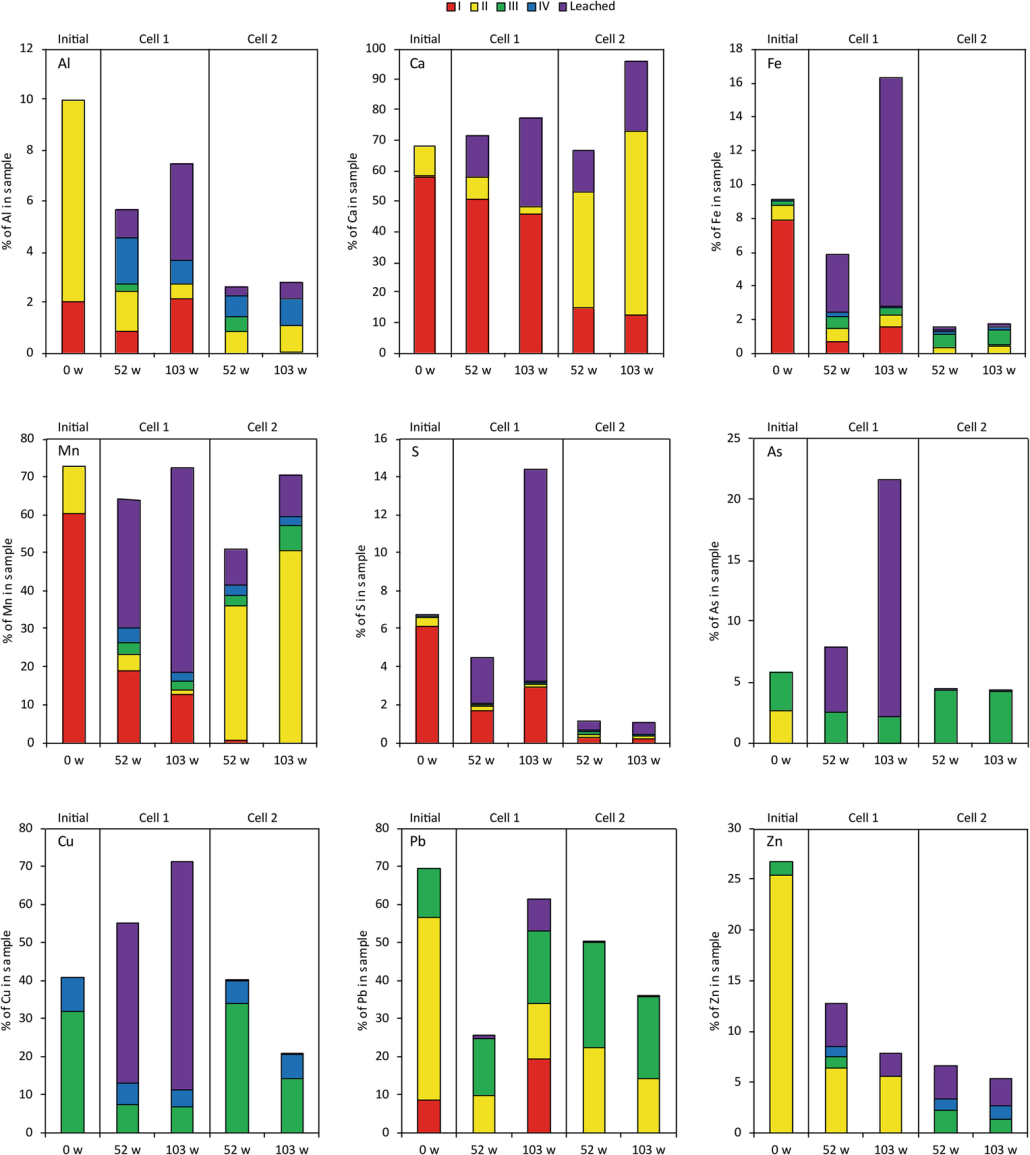
Extracted element content of the total element content in waste rock before leaching (initially) and from cell 1 (waste rock) and cell 2 (waste rock with 5 wt.% aged lime kiln dust) on separate occasions year 1 and 2 (weeks 52 and 103). Step I–IV in sequential extractions after Dold (2003). Water-soluble fraction (I), exchangeable fraction (II), Fe(III)oxyhydroxides (III), and Fe(III)oxides (IV). Concentrations below detection limit are not presented. Leached fraction is *estimated* based on element concentrations and withdrawn leachate volume

The initial waste rock was dominated by water-soluble compounds and oxidation products which typically would be stored in the pore water (step I) (Fig. 7). These compounds, such as melanterite and gypsum, are well known to precipitate in mine waste and are easily dissolved by, e.g., heavy rainfall (Nordstrom 2011). Elements which originated from sulfide oxidation and neutralization processes such as Fe, Mn, and S were released from both cells during the first weeks of leaching corresponding to an initial flush of weathering products (Fig. 6). Results from sequential extraction show a higher amount of elements such as Ca, Fe, Mn, S, and Zn in the water-soluble phase than was released during the first 3 weeks of initial flush of the waste rock which likely removed not only the fines but probably also a major part of the water-soluble compounds (Nyström et al. 2019). Following the re-sieving after 3 weeks of leaching the waste rock, the amount of water-soluble compounds decreased but was continuously present in the leaching of the waste rock. It is believed that the decrease in water-soluble was sped up by the sieving but that it would have occurred, even without the sieving, similar to an initial flush as indicated from the high concentrations observed during the first 3 weeks of leaching (Fig. 6). However, in Dold (2003), the water-soluble phase (step I) is determined by shaking the sample for 1 h in L/S 50. The weekly irrigation of the waste rock corresponded to an L/S of approximately 0.08, which was not enough to dissolve all water-soluble compounds during the first weeks which is in line with observations made by Maest and Nordstrom (2017).

Only approximately 75% of the added water volume was withdrawn on a weekly basis due to the capturing of water in the waste rock. When metal concentrations are high, such in cell 1, evaporation can lead to a build-up of oxidation products in the pore water shown as an increase of water-soluble compounds (Fig. 7).

**Fig. 8:**
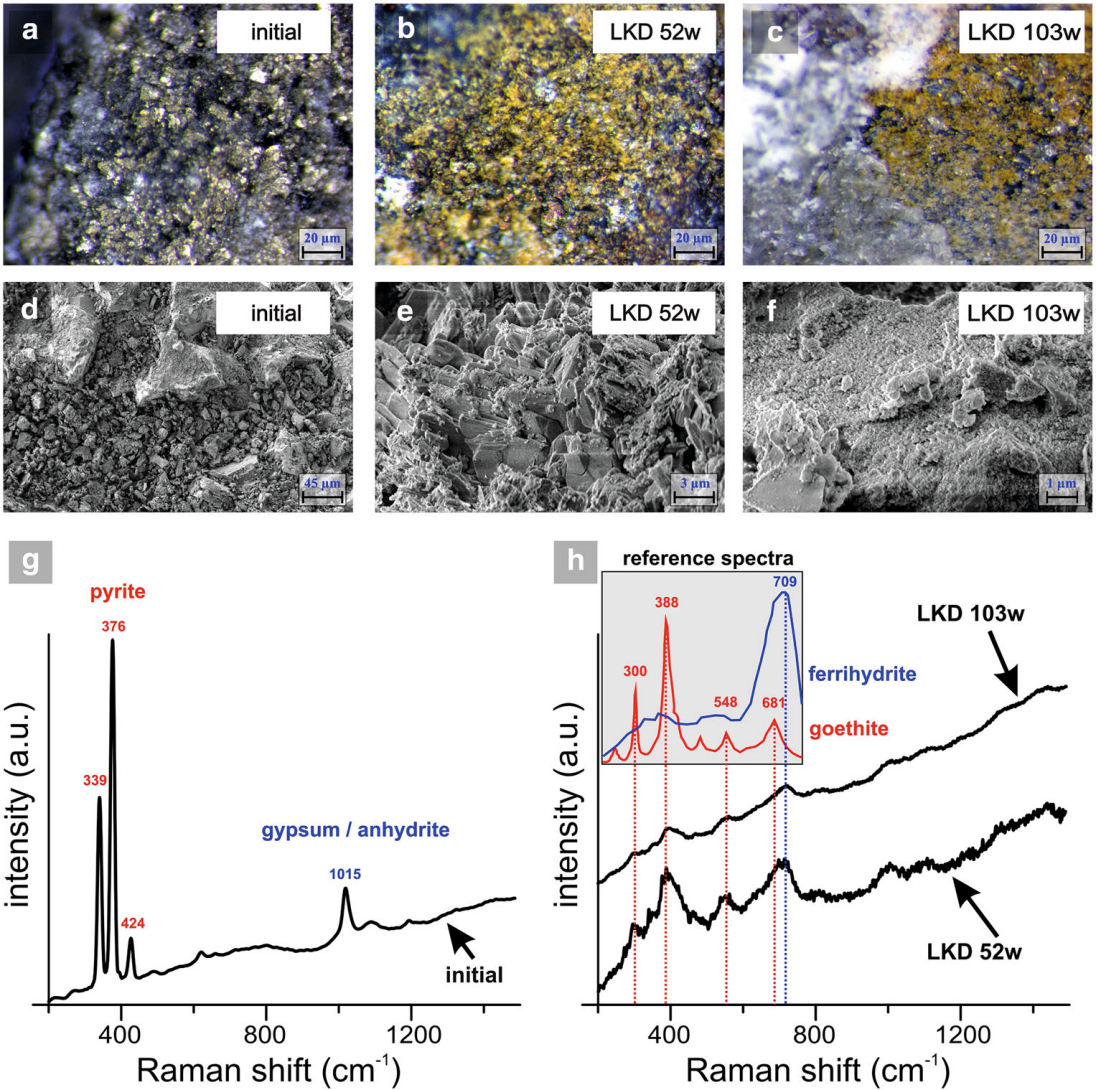
Stereomicroscope images (**a**–**c**) and secondary electron images (**d**–**f**) of waste rock samples before leaching (initially) and from cell 2 (waste rock with 5 wt.% aged lime kiln dust) on separate occasions years 1 and 2 (weeks 52 and 103). **g** Raman spectrum of one representative point in the initial waste rock showing the presence of pyrite and gypsum/anhydrite. **h** Representative Raman spectrum of waste rock with 5 wt.% lime kiln dust after 52 and 103 weeks of leaching showing the presence of goethite and ferrihydrite. Reference spectra were taken from Monnier et al. (2011)

Cell 2 showed an overall lower leachability of elements compared with cell 1 except for Ca and Mn which likely originated from the LKD addition. Lower leachate concentrations together with no observed acceleration of the sulfide oxidation in cell 2 suggest that a higher amount of metals and metalloids were released from the waste rock in cell 1 than in cell 2. Hence, HFO formation in cell 1 was prohibited by the low pH. Instead, more easily soluble phases (steps I–II) dominated. Opposite to cell 1, the amount of metals and metalloids released from the waste rock in cell 2 were able to precipitate as secondary minerals released by step III in sequential extraction. By coupling sequential extraction with geochemical calculations, these secondary minerals are suggested to conform of amorphous ferrihydrites such as 2-line or Fe(OH)_3_ (step III) along with more crystalline phases such as goethite and 6-line ferrihydrite (step IV) due to near-neutral pH conditions. Raman analysis of the samples taken initially and after 52 and 103 weeks of leaching in cell 2 shows a build-up of primarily goethite and ferrihydrite with increased coverage of the pyrite with time (Fig. 8) suggesting passivation of the sulfide surfaces. However, the main reason for a 99.9% decrease of, e.g., As, Cu, Pb, and Zn is believed to be the lack of accelerated sulfide oxidation owing to the LKD addition in cell 2.

Secondary minerals are crucial for the capture of trace elements (Petrunic et al. 2009; Jamieson et al. 2005), and HFO in particular are well known as important sinks for several metals and metalloids including As, Cu, Sb, and Zn (Essilfie-Dughan et al. 2012; Lalinská-Volekovová et al. 2012; Parviainen 2009; Alakangas and Öhlander 2006; Gunsinger et al. 2006; Foster et al. 1998). The addition of LKD led to a decrease in Zn, and it showed no immediate influence on As and Cu whereas the opposite was observed for Sb despite low content in the waste rock and low leachability of Sb from LKD (Fig. 6). Sb is known to form (hydr)oxyanions whose mobility is increased in circumneutral to alkaline waters due to decreased sorption on mineral surfaces such as HFO (Dzombak and Morel 1990). It is possible that some of the Sb was adsorbed to HFO forming during the first weeks of leaching and desorbed when LKD was added to cell 2, resulting in that the concentrations of Sb increased in cell 2 (Fig. 6). Similar behavior as Sb could be expected for As since (hydr)oxyanions are formed under these conditions, but no increase was observed. Instead, sequential extraction suggests a build-up of As associated with amorphous (oxy)hydroxides (step III) which could imply co-precipitation with HFO or other minerals. This means that, if the chemical environment was changed to more reducing conditions, for example during remediation, the secondary phases could be dissolved or transformed, releasing accumulated metals and metalloids adsorbed, co-precipitated, or present as a distinct mineral.

### Sulfur release and sulfide oxidation rate

**Fig. 9:**
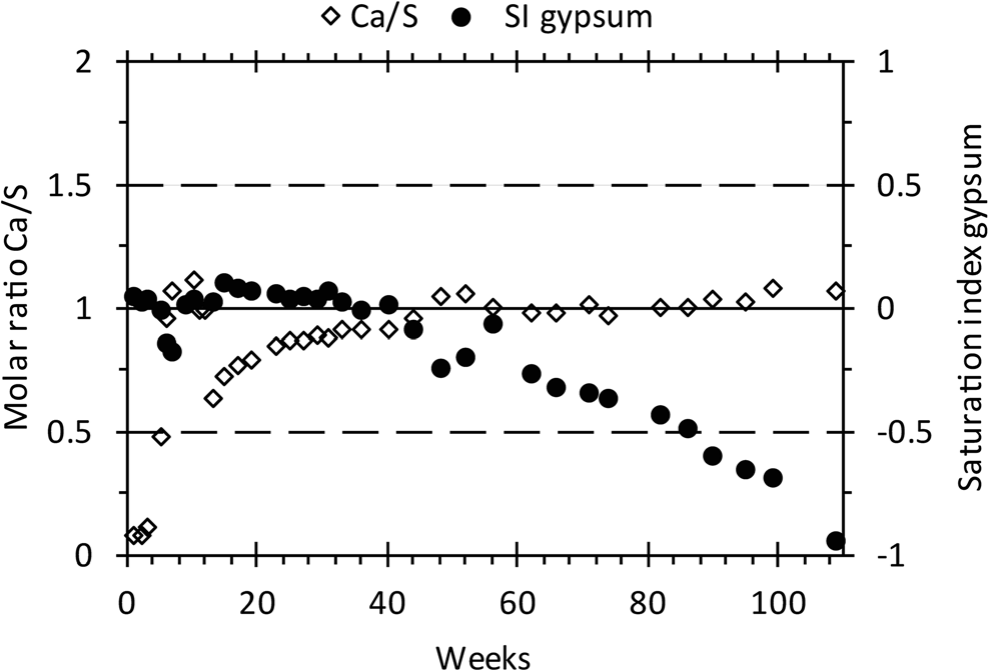
Molar ratio of Ca/S and saturation index of gypsum leaching of waste rock covered with 5 wt.% aged lime kiln dust (cell 2). Solid line illustrates the 1:1 relationship between Ca and SO4. Dashed lines mark values close to zero (± 0.5) which indicate equilibrium between the solution and mineral phase

Gypsum is one of the most abundant sulfate minerals and the most common sulfate salt in mining environment (Lottermoser 2010; Jambor et al. 2013) and often forms as a reaction between the oxidation and weathering products from the dissolution of pyrite (SO_4_) and calcite (Ca) (Plumlee et al. 2009). When sulfide oxidation is established, it is generally faster than calcite dissolution (Salmon and Malmström 2002) which often results in Ca being the controlling element in the formation of gypsum whose formation reduces both Ca and SO_4_ concentrations in the leachate and thereby can mask oxidation and weathering reactions occurring in the mine waste (Maest and Nordstrom 2017; Price 2009). However, due to the high solubility of gypsum, and its formation being pH-independent (Carroll-Webb and Walther 1988), it is challenging to lower SO_4_ concentrations in the leachate. Hence, the mechanism that removes SO_4_ from solution also causes elevated sulfate concentrations in the leachate (Lottermoser 2010). As seen in Fig. 6, the addition of LKD did not result in any immediate change in S concentrations. Despite the constant presence of S in the leachate, a notable decrease could be seen with time through the addition of LKD (cell 2). By examining the molar ratio Ca/S (Fig. 9), it was observed that S dominated over Ca at the beginning of leaching until week 44 when the ratio had increased to around 1. Simultaneously, the saturation index of gypsum started to decrease, although still within what can be assumed equilibrium between the solution and the mineral phase (Fig. 9). During the last weeks of leaching, gypsum became more undersaturated; meanwhile, the molar ratio of Ca:S remained around 1 with S being the limiting element which suggests that concentrations of S could originate from gypsum dissolution rather than sulfide oxidation. In general, the results suggest that the reason behind decreasing S concentrations is the formation of potentially long-term stable secondary minerals, such as goethite and ferrihydrite, that passivate the reactive sulfide surfaces (Fig. 8). However, in order to distinguish sulfide oxidation from the dissolution of secondary minerals, such as gypsum, which both can answer for, or part of, the sulfate release, it is crucial to estimate the oxygen consumption of the waste rock. No such information was included in the design of the experiment presented herein. However, it can be concluded that, regardless of the reason and long-term stability, an addition of a small amount of LKD to the waste rock had a positive influence on both the leachate quality and the sulfide oxidation rate resulting in an overall environmental improvement.

### Limitations

One concern with adding neutralizing minerals is that an excess of secondary minerals might form. In Sweden, regardless if inhibition and passivation of the sulfide surfaces are achieved or not, the waste rock will be backfilled into an open pit or dry covered. If an excess of secondary minerals is built up, latent acidity is stored over time, which may dissolve if the chemical condition changes to a more reducing condition. This can cause drainage from a pit lake or heap that will need treatment for an extended time before released into the recipient. The results obtained from this study suggest that the addition of LKD successfully prevents the sulfide oxidation and the subsequent release of metals and metalloids into the leachate. Moreover, the study suggests that the addition of LKD resulted in relatively low precipitation of secondary minerals compared with without addition. Also, not adding LKD to the waste rock will more likely generate drainage that needs treatment due to the dissolution of water-soluble minerals or adsorbed/exchangeable elements released when covered than if LKD is added to the waste rock as seen in cell 2. However, it cannot be excluded that there might be secondary phases forming on walls or at the bottom of the test-cell. Future research will focus on the identification of secondary minerals and trace element distribution in these.

A laboratory study by Strömberg and Banwart (1999) showed the importance of grain size, i.e., specific surface area, on the controls of acid neutralization due to carbonate dissolution. The favorable increase in the specific surface area suggests that carbonate-rich LKD can be advantageously compared, e.g., coarser crushed calcite. Even though it has been shown that the physical contact between the acid producing and neutralizing minerals is crucial for secondary mineral formation for passivation of the sulfide surface (i.e., mixing) (Pérez-López et al. 2007) applying the neutralizing material on top, or in layers in between, the acid-producing material can be advantageous due to costs and operational issues.

## Conclusions

The aim of this study was to evaluate the long-term (109 weeks) leaching of waste rock covered with a thin layer of LKD (5 wt.%) to determine the capacity of the LKD to maintain a near-neutral pH with the intention to inhibit the sulfide surfaces and thereby reducing the sulfide oxidation and subsequent release of sulfate, metals, and metalloids.

- Not treating the waste rock resulted in an acceleration of the sulfide oxidation and subsequent generation of ARD.
- The LKD is a rather pure material with high calcite content (85%), and its suitability for inhibition purposes, as described herein, was likely improved by storing the material.
- The addition of LKD effectively created and maintained a near-neutral pH throughout the time of leaching (109 weeks). Thereby, providing an optimal environment for precipitation of various Me-carbonates and hydroxides.
- The addition of LKD (5 wt.%) hindered acceleration of the sulfide oxidation which accounted for the majority of the difference in element release and lower sulfide oxidation rate compared with solely waste rock.
- Sulfur concentrations in the leachate decreased throughout the time of leaching; meanwhile, geochemical calculations of saturation index suggested gypsum dissolution, implying reduced sulfide oxidation rate in the waste rock.
- The decreased sulfide oxidation rate was probably due to passivation of the reactive sulfide surfaces by secondary mineral formation.
- Geochemical calculations suggested the formation of amorphous ferrihydrite such as 2-line or Fe(OH)_3_ along with more crystalline phases such as gibbsite and 6-line ferrihydrite. However, these minerals have yet to be identified but are expected to be responsible for the reduced sulfide oxidation rate and the attenuation of metals and metalloids such as As, Cu, Pb, and Zn either through adsorption, co-precipitation, or as their own mineral.
- If coatings on the reactive surfaces are not long-term stable, the formation of secondary minerals can lead to latent acidity through mineral dissolution if the chemical conditions change (by introducing, e.g., dry or wet cover). Therefore, any secondary minerals formed need to be characterized and assessed for their stability.
